# Tumor Bioengineering Using a Transglutaminase Crosslinked Hydrogel

**DOI:** 10.1371/journal.pone.0105616

**Published:** 2014-08-18

**Authors:** Josephine Y. Fang, Shih-Jye Tan, Zhi Yang, Charisse Tayag, Bo Han

**Affiliations:** Nimni-Cordoba Tissue Engineering and Drug Discovery Laboratory, Division of Plastic and Reconstructive Surgery, Departments of Surgery and Biomedical Engineering, Keck School of Medicine, University of Southern California, Los Angeles, California, United States of America; University Hospital of Modena and Reggio Emilia, Italy

## Abstract

Development of a physiologically relevant 3D model system for cancer research and drug development is a current challenge. We have adopted a 3D culture system based on a transglutaminase-crosslinked gelatin gel (Col-Tgel) to mimic the tumor 3D microenvironment. The system has several unique advantages over other alternatives including presenting cell-matrix interaction sites from collagen-derived peptides, geometry-initiated multicellular tumor spheroids, and metabolic gradients in the tumor microenvironment. Also it provides a controllable wide spectrum of gel stiffness for mechanical signals, and technical compatibility with imaging based screening due to its transparent properties. In addition, the Col-Tgel provides a cure-*in-situ* delivery vehicle for tumor xenograft formation in animals enhancing tumor cell uptake rate. Overall, this distinctive 3D system could offer a platform to more accurately mimic *in vivo* situations to study tumor formation and progression both *in vitro* and *in vivo*.

## Introduction

Tumors are dynamic and complex structures. Their composition and environment are governed by biochemical and molecular signals exchanged between cells and their extracellular matrix (ECM) [1], [2]. Even though 2D tumor cell cultures have been used routinely for conducting biochemical and drug sensitivity tests in oncology, they seldom mimic the *in vivo* environment, and scarcely reflect integral biomimetic characteristics such as cell-cell and cell-matrix interactions and their corresponding spatiotemporal signaling, metabolic gradients, and mechanical restriction [3]–[5]. Thus, bioengineering tumors by using biological relevant 3D tumor cell culture models can bridge between *in vitro* cell based assay and the native microenvironment of living organisms [6]–[8]. In addition, 3D culture systems generated from human tissue could be a better tool for drug screening by implementing more accurate *in vivo* equivalent structures and organization and might produce more predictive response than non-human systems [9].

Many 3D tumor cell culture models ranging from scaffold-dependent to scaffold-free, and consisting of single or multiple cell types have been developed. These models provide the opportunity to simulate important aspects of tumor masses including cancer cell aggregation and clustering, cell migration and proliferation, angiogenic factors release and hypoxia [10]. One of the most widely used models is the Multicellular Tumor Spheroids (MCTS) system, a scaffold-free tumor cell system that can facilitate cell-cell interactions through chemical linkers or gravitational enhancement [7]. Many extracellular matrices (ECM) such as Matrigel, type I collagen, fibrin, and hyaluronic acid have been used as tumor cell 3D scaffolds [11]–[13]. These biologically derived matrices provide both chemical and mechanical cues essential for modulation in gene expression while allowing for cellular adhesion and integrin engagement [14]–[18]. However, there are still some incomplete requirements for cancer research and drug development, such as unknown dose of growth factors and additives in the preparations, uncontrollable mechanical rigidity, batch to batch variations, low reproducibility, complex protocol setup, and physiological irrelevant matrices for cells.

The ECM plays an important role in supporting or even inducing tumorigenesis [7], [8]. The most common extracellular matrix component presenting in the tumor microenvironment is collagen, which provides a scaffold for structural support. Meanwhile, collagen turnover in the tumor microenvironment was associated with tumor progression and metastasis [2]. In previous studies, we have developed an injectable gelatin-based transglutaminase-crosslinked gel system (Col-Tgel) for cell culture and drug delivery [19]–[21]. Here we focus on the development and validation of novel 3D culture system that simulate the tumor stromal environment by manipulating the Col-Tgel. We demonstrated that biocompatibility and 3D architecture of Col-Tgel were suitable for reproducing the solid tumor microenvironment and it may offer a toolbox to study key events associated with tumor formation, progression, and metastasis and have potential to serve as an antitumor drug testing platform [22]–[24].

## Materials and Methods

### Cell culture

MDA-MB-231 (human breast carcinoma), Saos-2 (human osteosarcoma), and HCT116 (human colorectal carcinoma) cell lines were obtained from ATCC (Cat.HTB-26, HTB-85, CCL-247, American Type Culture Collection, Manassas, VA). The C4-2B human prostate cancer cell line was generously provided by Dr. M. Stallcup and SCC-71 human oral squamous carcinoma cell line was gifted from Dr. Uttam Sinha (Norris Cancer Center at USC) [25], [26]. MDA-MB-231, Saos-2, SCC-71 were first expanded in traditional 2D culture in DMEM, HCT116 in McCoy5a, and C4-2B in RPMI1640 (Mediatech, VA), all with 10% fetal bovine serum (Lonza, MD) supplement and 1% Penicillin/Streptomycin (Mediatech, VA). Rat bone marrow derived mesenchymal stem cells were prepared in our laboratory as described [27], [28].

### Gel preparation and characterization

Transglutaminase-crosslinked collagen hydrogels (Col-Tgel) were prepared as described previously [29]. Briefly, 12% gelatin (bovine skin type B 225 bloom, Sigma- Aldrich, MO) was prepared with 2× PBS and autoclaved for sterilization. 4°C stored stock gel was liquefied at 37°C and further diluted to 6% with dH_2_O. Diluted gel was handled at room temperature for all assays and cell embedding.

Light transmission of Col-Tgel, compared with type I collagen 3 mg/ml (BD Bioscience, CA) and Matrigel with phenol red free (BD Bioscience, CA) was measured in 1ml cuvette with wavelengths of 600 nm using a UV visible spectrophotometer (Hitch U-3000, Japan). The higher absorbance value represented the lower transparency of the gel.

Mechanical test were carried out with an indentation test. Gelatin gel with concentrations of 3, 4.5, 6, 7.5 and 9% was prepared and 3 ml of gel was loaded in a glass tube sample container. After gel polymerized, the gel surface was marked as initial height followed by gently applying a 5.8 g and 8 mm diameter stainless steel sphere. The sphere was placed at the centre of the sample and the weight of the sphere caused the gel deformation. The side-view image of the gel deformation was recorded by mounted camera with a reference ruler. However, the ratio of the gel height and the distance of indentation was not less than 10% and the ratio of the gel lateral dimension and contact radius was not higher than 12. Therefore, the half-space assumption and Hertz contact theory cannot apply in this circumstance [22]. The central deformation of the gel construct cannot be directly used to compute the Young's modulus, so we simplified demonstrate the displacement distance as an indicator of relative gel stiffness in this study.

### 3D Col-Tgel culture

**Figure 1:**
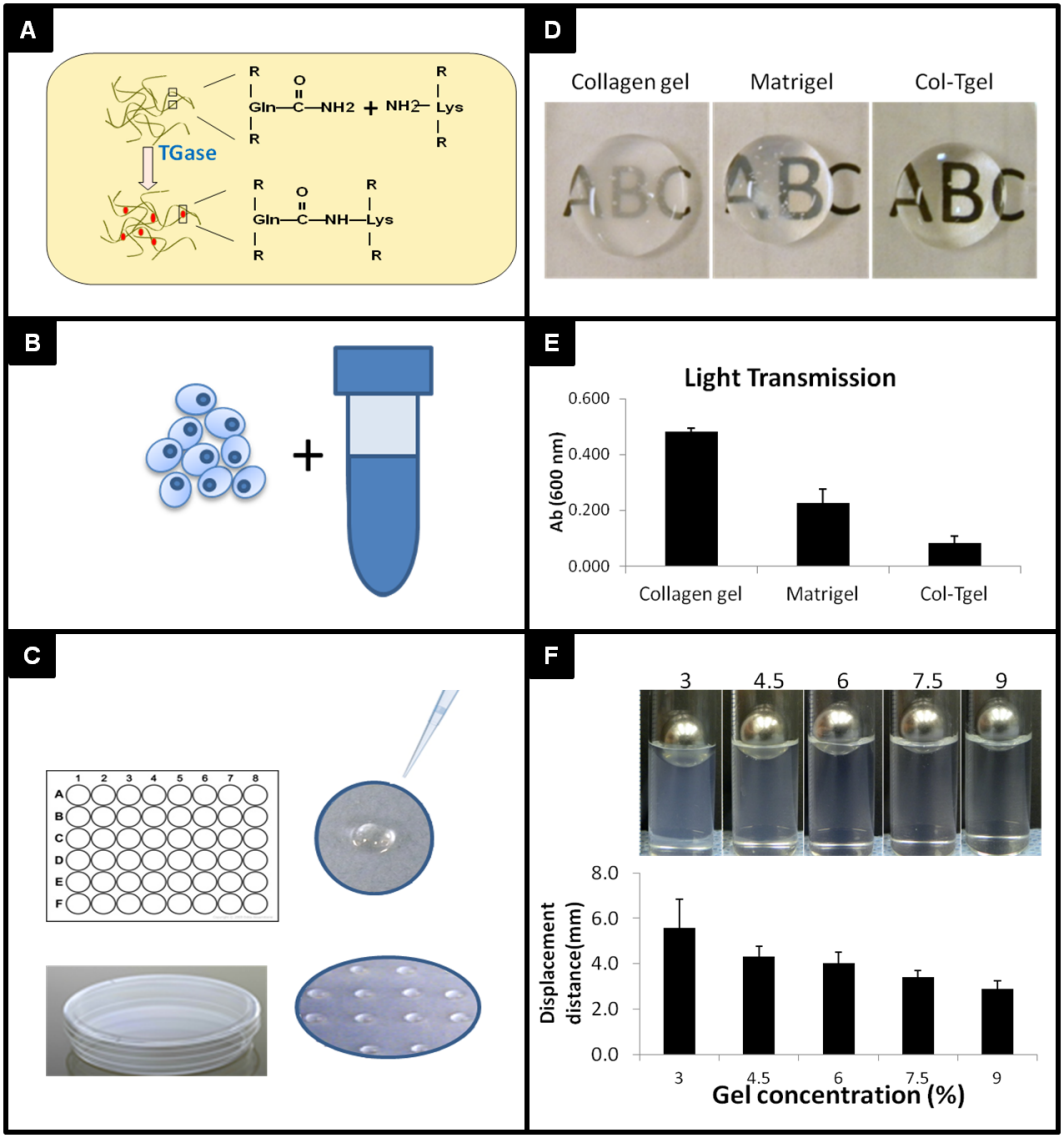
Schematic illustration of 3D Col-Tgel tumor model. A, Enzymatic crosslinking of collagen base gel by transglutaminase. B, Tumor cells suspended in soluble gel at desired densities. C, Gel was pipetted into a single well as a droplet, or multi droplets in single dish after addition of crosslinker, Tgase. D&E, Comparison of transparency of three different 3D matrices, Type I collagen, Matrigel, and Col-Tgel. F, The stiffness of Col-Tgel was manipulated by changing the gel concentrations.

Cells were trypsinized with 0.025% trypsin in HBSS (Mediatech, VA) washed with 1XPBS and counted. A calculated volume of gelatin solution was added into the cell pellet to make desired final seeding density (Fig. 1). Cells were suspended in the gelatin solution followed by addition of purified transglutaminase (Tg) crosslinker for final concentration of 50 µg/ml by gentle pipetting [30], [31]. Cell-seeded hydrogel was placed as a single droplet on the surface of a 48-well suspension cell culture plate or multiple droplets on 4 mm dishes (Greiner bio-one, NC) following by incubation at 37°C for 30 minutes. The volume of the gel-cell suspension was varied to fabricate the specific radius of Col-Tgel-cell dome. Cell type specific medium was added for prolonged culture.

### 3D morphological analysis

Cells in 3D gel were directly observed daily under the light microscope (Leitz Wetzlar, Germany) and recorded with a digital camera (Nikon, Japan). The center-to-edge images were recorded using a light microscope by adjusting the focus plain on the z-stack.

For immunohistochemical analysis, cells in 3D gel were fixed with 10% neutral formalin for 10 minutes. After washing with PBS, cells were permeabilized with 0.5% Triton X-100/PBS, and blocked with 3% bovine serum albumin for 30 minutes at 37°C following by incubation with fluorescence protein conjugated primary antibody Rhodamine Phalloidin 33 nM (Cat.R415, Lifetech, CA) and DAPI dihydrochloride (dilution = 1∶100000, Cat.40011, Biotium Inc., CA) (nuclei staining) in the dark for 5 minutes. In addition, other polyclonal rabbit primary antibodies, such as E- cadherin (dilution = 1∶400, Cat.PA5-32178, Thermoscientific, IL), laminin 5 (dilution = 1∶400, Cat.ab14509, abcam, MA), β1 integrin (dilution = 1∶400, Cat.PA5–29606, Thermoscientific, IL) were incubated with 3D culture constructs overnight at 4°C and detected with FITC conjugated goat antibody (dilution = 1∶1000, Cat.65–111, Lifetech, CA). Imaging was taken by EVOS fluorescence microscope (Advanced Microscopy Group, WA) directly in culture wells.

For immunocytochemistry, the samples were proceed with the same procedure as above and incubated with polyclonal rabbit primary antibody Ki67 (dilution = 1∶100, Cat.PA1–21520, Thermoscientific, IL), anti-rabbit IgG-biotin antibody from goat as secondary antibody (dilution = 1∶600, Cat.B8895, Sigma-Aldrich, MO), streptavidin-peroxisase polymer as signal amplifier (dilution = 1∶800, Cat.S2438, Sigma-Aldrich, MO) and detected by Pierce Peroxidase Detection Kit (Cat.36000, Thermo scientific, IL). MSC and SCC-71 were labeled with Mitotracker green and red (Cat.M7512, Lifetech, CA) respectively before co-culture.

### Cell viability and gel permeability

Cell viability for the encapsulated cells was quantified by Cell Counting Kit-8 (Cat.CK04–13, Dojindo Laboratories, Japan) and followed by manufacturer's protocol. Dehydrogenase activity from live cells reduces red tetrazolium component into a soluble yellow formazan in the medium, which is directly proportional to the number of living cells. Briefly, at predetermined time points, media was replaced with 200 µl of fresh media containing 5µl of stock CCkit8 reagent. After three hours of incubation, 100 µl of medium was transferred into a 96-well plate for 450 nm absorbance measurement by multiplate reader (Molecular Devices, CA). Reagent contained medium without cell incubation served as baseline control.

For viability assay and gel permeability tests, cells embedded in Col-Tgel were treated with 5 mg/ml of 3-(4, 5-Dimethylthiazol-2-yl)-2, 5-diphenyltetrazolium bromide (MTT) for 3 hours. The reaction was stopped by adding DMF/SDS (pH 4.7). The reduction of an MTT tetrazolium component into an insoluble dark purple formazan by viable cells was imaged by light microscope.

### Oxygen level detection

MDA-MB-231 cells were embedded in a 100µl Col-Tgel droplet at 1×10^6^/ml and cultured in DMEM with 10% FBS and 1% PS at 37°C. Oxygen concentration was measured by OM-1 Oxygen meter (Microelectrodes Inc, NH) probed at the gel center and in the surrounding medium at day 0, 3 and 9.

### Cancer drug testing in a 3D model

Each 20 µl gel droplet with 4×10^4^ cells was cultured in a 48-well-plate for 3 days before drug treatment. Paclitaxel (TEVA Pharmaceuticals Inc., USA) in final concentrations of 0, 2, 20, and 200 µM were added. For comparison, the same numbers of cells as used in monolayer cultures were treated with the same drug regime. After 72 hours, cell viability assay by CCkit-8 (Dojindo Laboratories, Japan) together with Live/Dead cell assay (Cat.L3224, Lifetech, NY) were performed. Briefly, 3D culture were incubated for 30 minutes in media containing 4 mM calcein AM (λem = 530 nm) to stain viable cells and 1.5 mM ethidium homodimer-1 (λem = 645 nm) to stain dead cells.

### Tumor Induction in nude mice

The animal surgery procedures and housing conditions in the study was followed by the approved protocol from Institution of Animal Care and Use Committee (IACUC) at University of Southern California and permission number is 11732. All animals were handled under aseptic condition at all time during and after the surgery. Food and water were given ad libitum throughout the study. Total 36 of 6 week-old and ±22 g male athymic nude mice (Simonsen Laboratories) were used as xenograft hosts. Animals were divided into six groups for six type of cancer cell line induction. Briefly, 100 µl Col-Tgel containing 1×10^6^ cells of each tumor cell type (MDA-MB-231, HCT116, CFPAC-1, and SaoS-2) was subcutaneously injected into both flanks of mouse after anesthetizing with Ketamine/Xylazine. Tumor sizes were measured every three days. Retrieved tumor specimens were processed for pathological assays.

### Statistic analysis

Experiment results were analyzed with nonparametric ANOVA by SAS (SAS institute Inc.) due to the small amount sample size. Experiment data were converted to rank transformation and analyzed correlation and regression of each study group with rank transformation by PROC GLM. Since sample size were the same, we choose Tukey's test for pairwise comparison (p<0.05 for drug resistance test, p<0.001 for oxygen level test).

## Results

### The collagen derived 3D gel for engineering tumors

Col-Tgel containing native and denatured type I collagen peptides provides an extracellular environment for cell attachment and growth [23], [32] (Fig. 1A). Before crosslinking, gelatin maintains liquid phase at room temperature for homogenously mixing with cells (Fig. 1B). After adding of crosslinking enzyme, 3D gel could be formed in about 30 minutes at 37°C. This time window was allowed to transfer and modify gel-cell constructs either as a single drop or multi-gel droplets into each culture compartment (Fig. 1C). The dome height on the hydrophobic surface is correlated to the cast volume. After incubation, Col-Tgel transformed from liquid into irreversible hydrogel to encapsulate cells in situ. The cured Col-Tgel is transparent at all tested concentrations as compared to semi-opaque type I collagen gel and Matrigel, as shown by opaqueness comparison images (Fig. 1D) and light transmission measurement (Fig. 1E). This property enables to study live cell morphology change, cell-cell interaction, and migration simply under the optical microscope. Col-Tgel also exhibited variable mechanical stiffness property with the alteration of gelatin concentrations and crosslinking rate. In general, the stiffness increases as the concentration of gelatin increased, where 9% Col-Tgel presented the highest stiffness while 3% was the lowest as illustrated by indentation technique (Fig. 1F). This is an important property of the Col-Tgel, indicating gel restriction force would be directly manipulated by controlling the gel concentration to generate interstitial pressure applied on cells.

**Figure 2:**
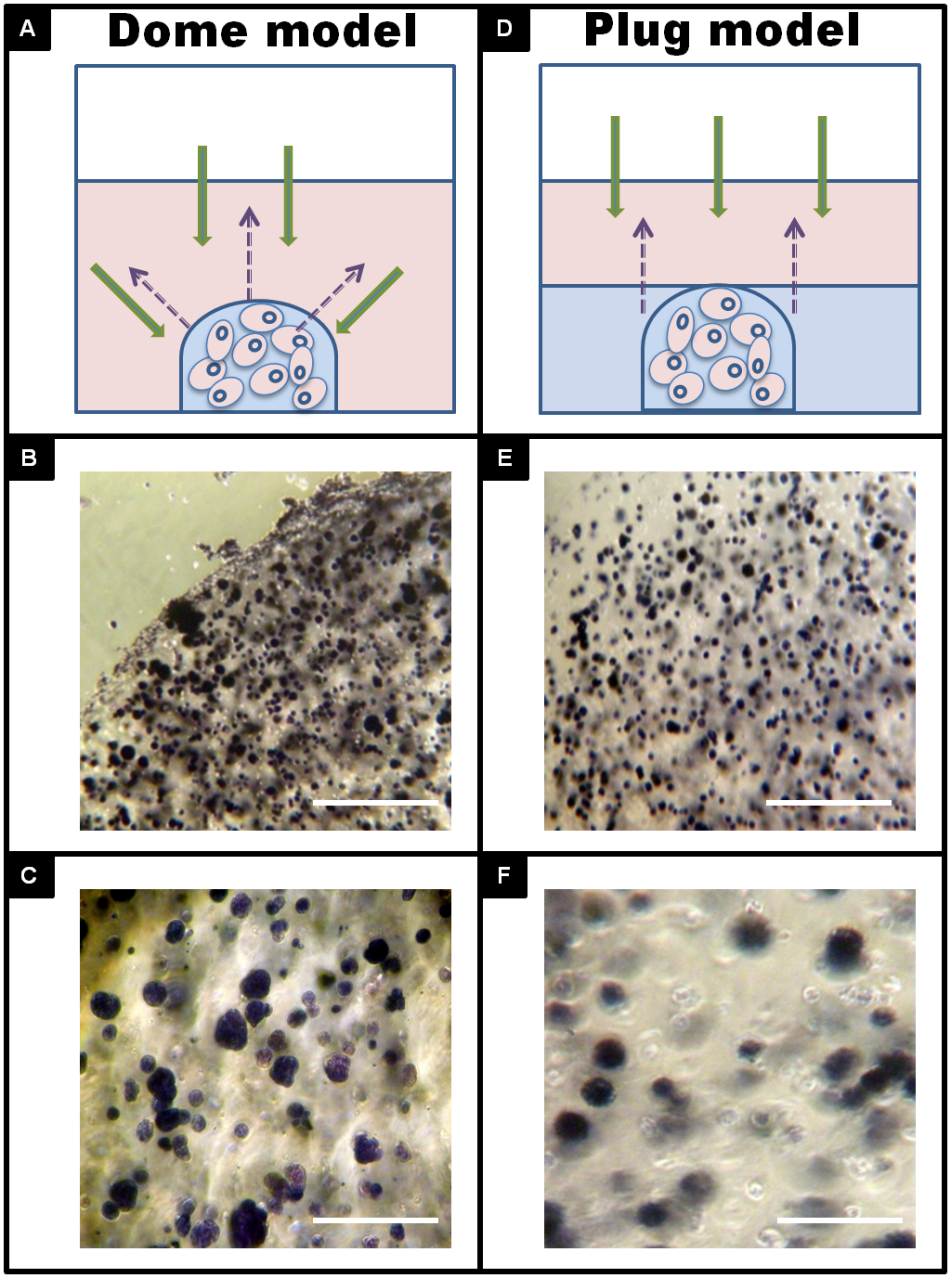
3D *in vitro* tumor models. A, Dome model, 20 µl of gel was pipetted on the bottom of each well of 48-well plate. D, Plug model, on top of the dome, 100 ul of gel was added to fill to the height of the dome. MTT diffusion and reduction by MDA-MD-231 cells was monitored by inverted light microscope after 3 hours addition. Micrographs were obtained under light microscope on the edge (B) and center (C) of dome model or surface (E) and bottom (F) of plug model. Scale bar = 500 µm for fig. 2B and 2E, scale bar = 100 µm for fig. 2C and 2F.

Matrix geometry is one of the critical factors in determining cell fate [33]. To further define the 3D culture system, we created two 3D culture models in the multi-well plate and tested the gel geometry effects on nutrient permeability: a dome model (Fig. 2A) and plug model (Fig. 2D). We generated one dome per well in 48-well plate. In plug model, a layer of gel was further casted on top of cured dome to ensure the initial cell number and their positions were the same. MTT was served as a sample molecule to study the gel shape effects. Diffused MTT was absorbed by cells, and reduced into insoluble purple formazan crystals. After three hours, all MDA-MB-231 cells in the dome model turned purple regardless of cell location (Fig. 2 B&C). However, color changes in the plug model were cell location dependent, purple on the top surface (Fig. 2E) while partially stained cells at the bottom (Fig. 2F). Due to the fact that dome shape ensures reproducible analysis and roughly identical radial diffusion barriers towards the centre of the dome, the dome model was selected for the succeed studies.

### Formation of tumor spheroids in 3D gel

**Figure 3:**
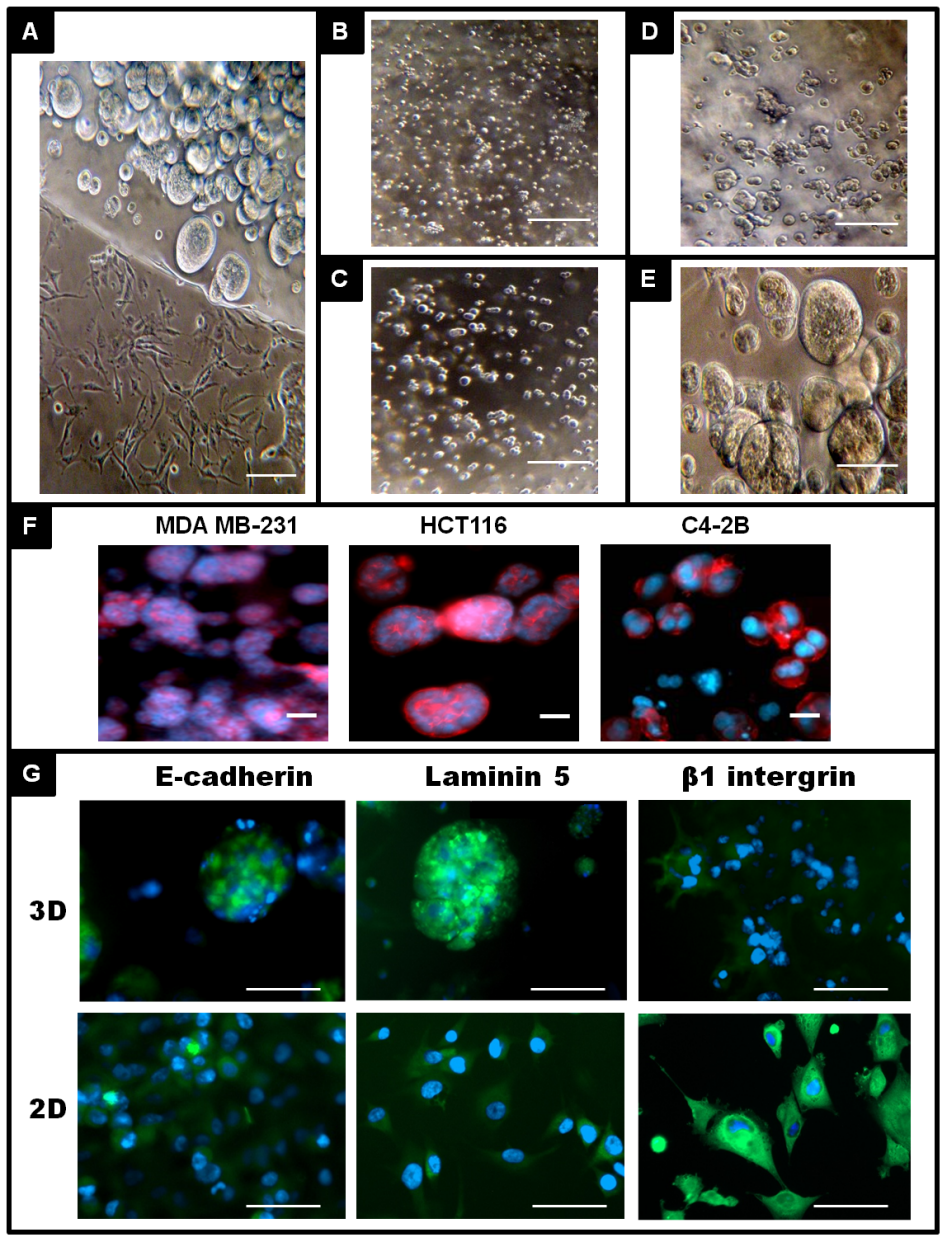
Formation of tumor spheroids in 3D gel. A, Breast cancer cells (MDA-MB-231) were culture in 3D dome gel for 6 days and formed spheroids inside the gel. Cells started to migrate out of gel either as individual cell or as collective cell clusters. Migrated cells showed elongated branched morphology on 2D surface. B–E, Time course of spheroid formation (scale bar = 140 µm). MDA-MB-231 M cells were cultured in 3D Col-Tgel for 0, 2, 4, and 6 days and recorded observation under light microscope (B–E, scale bar = 140 µm). F, Prostate cancer C4–2B cells, Colon cancer HCT116 cells and Breast cancer MDA-MB-231 cells were stained with F-actin (red) and DAPI (blue) and observed under fluorescence microscope (scale bar = 100 µm). G, MDA-MB-231 cells cultured in 2D or 3D were IHC stained with E-cadherin, laminin 5 and β1 integrin (scale bar = 100 µm).

Col-Tgel serves as an interstitial substrate to support the growth of different cell types including tumor and normal cells. The transparency of Col-Tgel enabled us to observe cell assembly in real time. Tumor cells adopted unique morphology when cultured in Col-Tgel 3D in comparison to 2D plastic surface. MDA-MB-231 cells developed self- assembly microspheroids inside Col-Tgel after 6 days of culture (Fig. 3A). Cells aggregated, formed tight cellular clusters ranging 30 to 200 µm in diameter, and shaped similar to *in vivo* tumors (3A, top, inside gel). In comparison, the same MDA-MB-231 cells growing on a plastic surface (3A, bottom half, on plastic) presented a completely different cellular morphology in the same culture well. Cells exhibited elongated morphology with pseudopodium protrusion. There was little cell-cell junction formation between neighboring cells.

**Figure 4:**
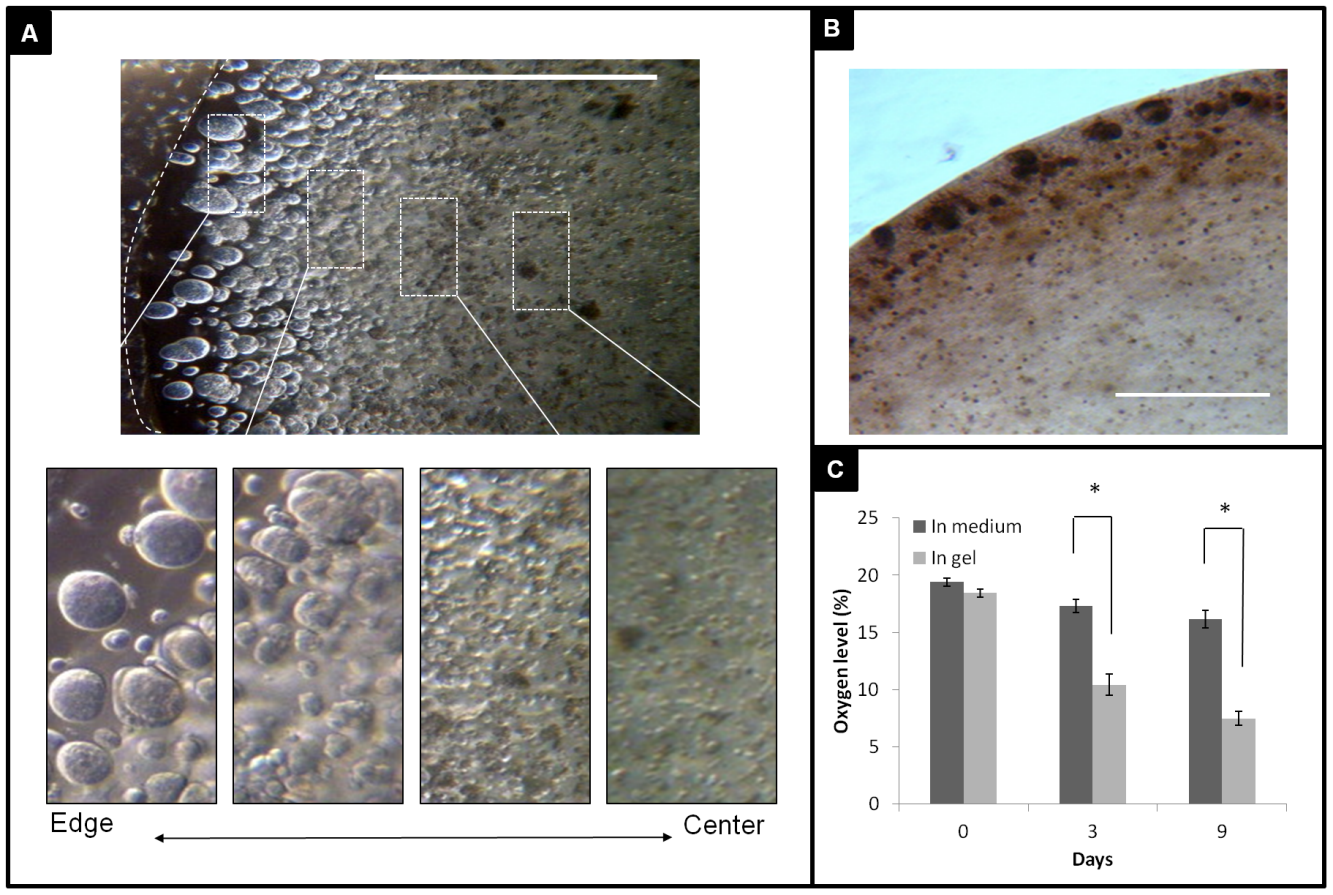
3D tumor model reproduce tumor cell heterogeneous conditions. A, Tumor cells formed quiescent or necrotic center; small to big spheroids from the innermost to the periphery region of the gel when MDA-MB-231 cells were cultured for 6 days (scale bar = 1000 µm). B, MDA-MB-231 cells stained with proliferation marker anti-Ki67 antibody at day 6 and showed strong to faint staining from edge to center (Scale bar = 300 µm). C, Oxygen concentrations were measured in the medium and in the gel. OM-1 oxygen meter probe was used to directly measure the O_2_ percentage in medium and in gel. *P<0.001, data mean ± standard deviation, n = 4.

The time course of spheroid formation is shown in (Fig. 3B–E). On initial seeding, cells were homogenously distributed throughout the gel with no aggregates or clusters formation (Fig. 3B). After 48 hours, 2- to 3-cell aggregates formed (Fig. 3C). Using a time-lapse camera, initial cell clusters were formed by both cell multiplication and assembly (data not shown). After four days in culture, cell clusters grew bigger with 10 to 20 cells in each cluster (Fig. 3D). By six days, a large number of spheroids aligned around the periphery of the gel dome. Spheroids contained ten to hundred cells and their shapes from round to oval with smooth surfaces (Fig. 3E). Other tumor cells such as prostate cancer cells (C4–2B), and colon cancer cells (HCT116) presented similar cell arrangements in 3D Col-Tgel (not shown). When stained with the cytoskeleton protein, F-actin, spheroids displayed distinctive cell morphology in different tumor cells (Figure 4F, prostate cancer C4–2B, colon cancer HCT116, and breast cancer MDA-MB-231) [34].

The surface expression of E-cadherin, laminin 5 and β1 integrin was comparatively analyzed in 2D monolayer cells and 3D spheroid in MDA-MB-231 cell line (Fig. 3G). When MDA-MB-231 cells growing in 3D Col-Tgel, cells spontaneously formed into spheroids and exhibit positive E-cadherin staining, which was resemble the morphology of xenograft derived from the same cell line and localized on the cell-cell contact surfaces within the clusters in the *in vivo* study. However, MDA-MB-231 cell expressed relatively less E-cadherin when cultured on the 2D monolayer even cells reached confluence. In addition, the cell-matrix adhesion molecule laminin was also expressed intensively by spheroid type of cells, and located on the outer layer of the spheroids. Again, laminin expression was reduced in the single cell and 2D culture. In contrast to E-cadherin and laminin, β1 integrin was expressed abundantly in the monolayer culture, but relatively less in the 3D culture. High level β1 integrin expression on cells revealed mesenchymal morphology but attenuated in the epithelia round shaped cells. These results suggested that cell morphology alteration in monolayer and 3D environment were dependent on extracellular matrix 3D microenvironment by accompanying with a loss or aberrant expression of adhesion molecules.

### Heterogeneous tumor model inducing hypoxia

After culture in the 3D gel for a few days, cancer cells on the periphery were seen to actively proliferate and developed spheroids while the innermost ones remained quiescent or necrotic (Fig. 4A and inserts). The sizes of tumor spheroids decreased from edge to center. When the distance is over 1000µm from edge, the cells were less proliferative as shown by active proliferation marker Ki67 staining (Fig. 4B). A large number of dead cells presented in a large void with no visible cell structures. To examine if this phenomenon resulted from decreasing nutrient diffusion and deprivation of oxygen at the dome center, we measured the oxygen concentrations using an oxygen probe. We found there was no difference in oxygen levels between gel and medium on the initial day. With increasing culture time, oxygen levels in Col-gel were lower than surrounding medium, and dropped significantly with the culture time (Fig. 4C). A hypoxic environment was observed around actively proliferating cells which voraciously consumed oxygen while forming a cellular barrier to prevent oxygen diffusion to the center. This feature was correlated with the avascular and hypoxic tumor microenvironment seen in *in vivo*.

### Co-culture of cancer cells and mesenchymal cells to mimic the tumor niche

**Figure 5:**
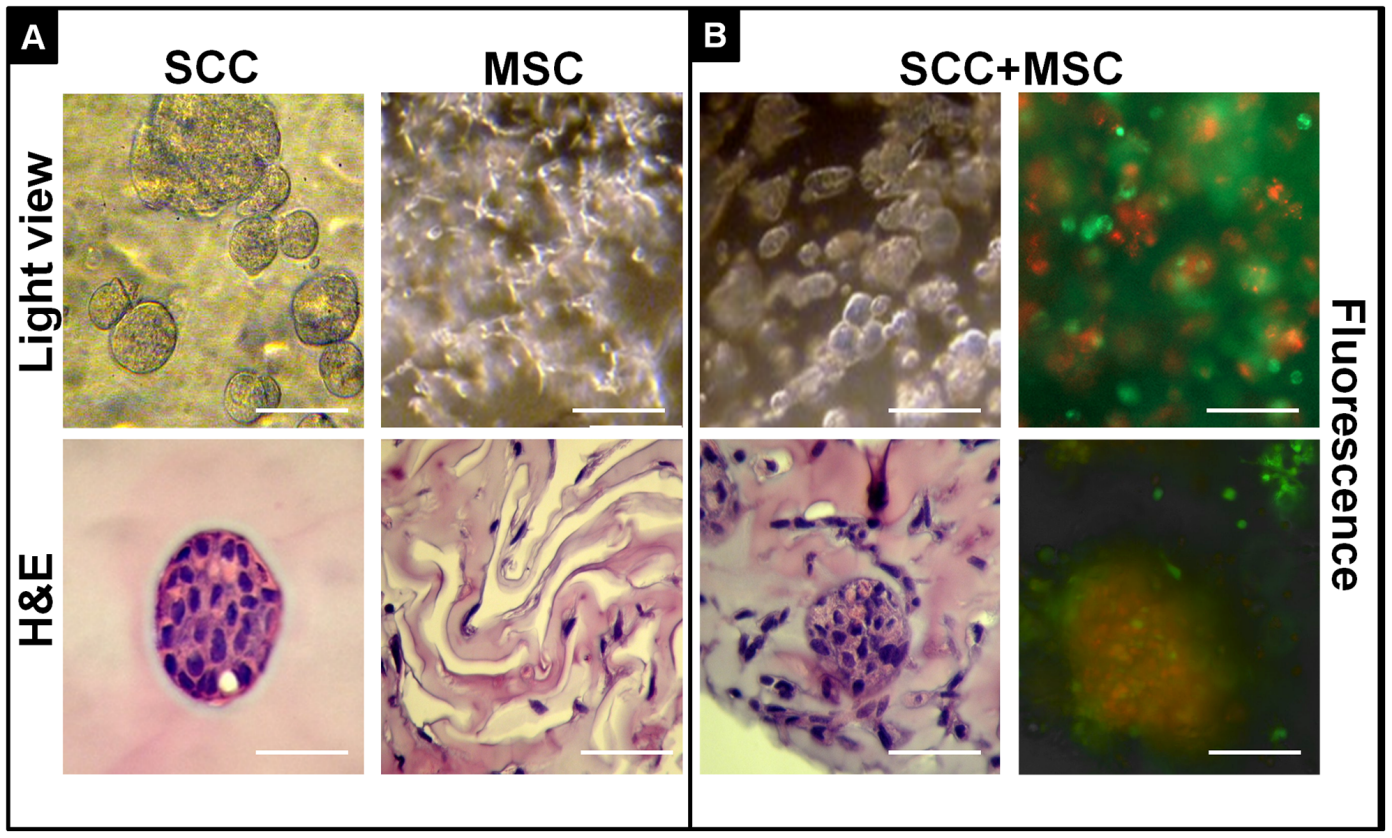
Tumor cells and mesenchymal cells showed different cell-cell and cell-matrix interaction in 3D Col-Tgel system. A, Individual culture of human squamous cell carcinoma line, SCC-71 and bone marrow derived mesenchymal stem cells in 3D gel for 6 days. B, Chimera tumor spheroid were formed when co-culture of SCC-71 and bone marrow derived mesenchymal stem cells in 3D Col-Tgel system for 6 days. Top row: light microscope; bottom row: H&E staining from paraffin section. Fluorescence: MSC (green) and SCC-71 (red). Scale bar = 100 µm.

*In vitro* tumor models can be made more relevant by recruiting two or more cell types to study cell-cell interactions and cell-matrix interaction. A human squamous cell carcinoma line, SCC-71 and bone marrow derived mesenchymal stem cells were cultured separately or co-cultured together in the 3D Col-Tgel system (Fig. 5). SCC-71 cells formed spheroids in Col-Tgel after day 6. The multi-cell spheroids displayed borders in the gel without visible digestive rings. On the other hand, bone marrow derived mesenchymal stem cells exhibited another exclusive morphology in the gel. After 6 days of culture, cells presented stellate structure and seemed to dig tunnels appearing with light reflection in the gel. H&E staining of MSCs showed cells were separated from each other with elongated shape and degraded the surrounding gel environment leaving multiple clefts. When SCC-71 cells were co-cultured with MSC cells mixing homogenously at 1∶1 ratio on day 0, after 6 days, both cells maintained their distinctive morphologies. SCC-71 cells formed clusters with tight junctions while MSC cells kept as individual elongate shapes surround the tumor spheroids. When two types of cell were labeled with different colors of fluorescence (MSC green and SCC-71 red), chimera spheroids formation in orange color was observed. The exact dynamic and structure of chimera spheroids formation requires further characterization. Collectively, the Col-Tgel can create a tumor microenvironment to study cell-cell interaction between cancer cells and their associated cells in 3D model.

### 3D model for drug test

**Figure 6:**
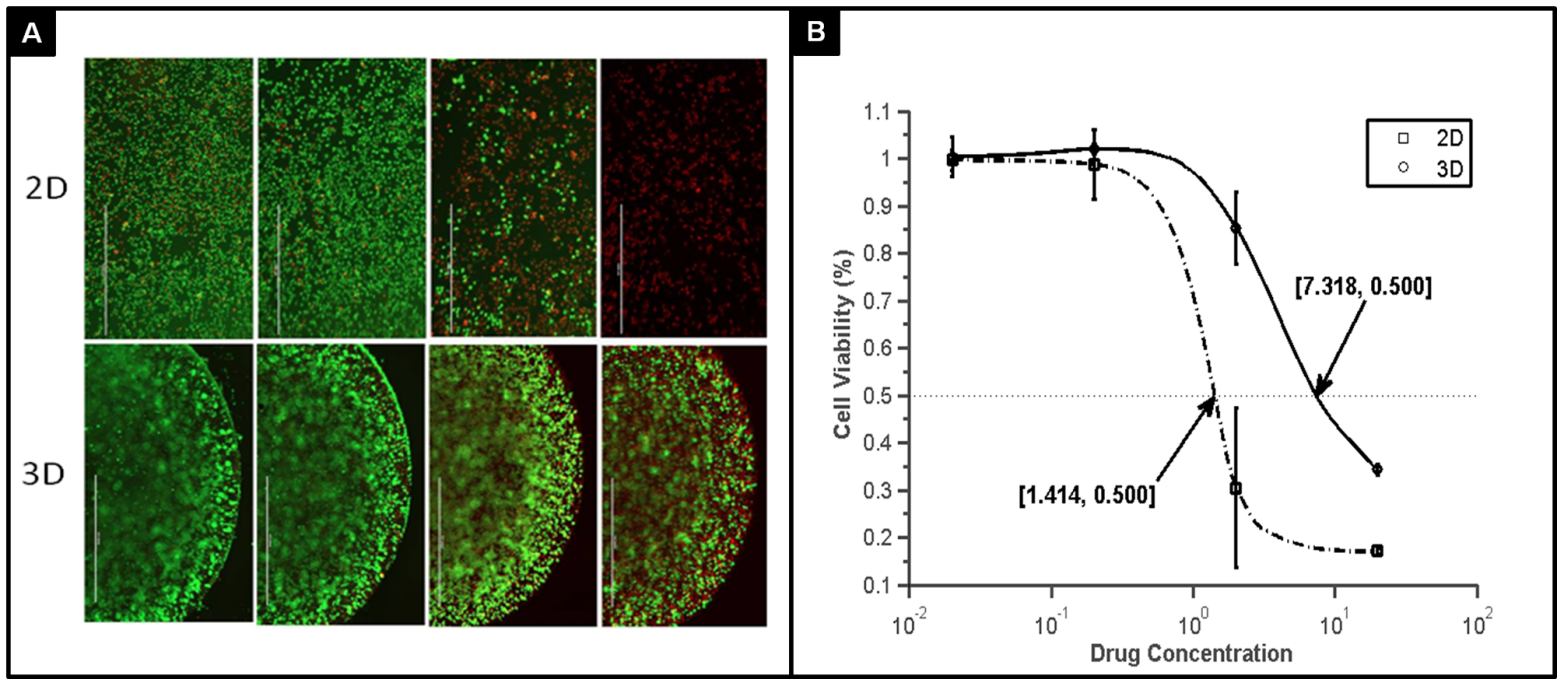
Drug sensitivity test on MDA-MB-231 cells in 2D and 3D. A, MDA-MB-231 cells were treated with paclitaxel at concentrations of 0, 0.2, 2, and 20 µM (from left to right, scale bar = 2000 µm) and analyzed with Live/Dead cytotoxicity/viability kit. Dead cells were in red and live cells in green. B, MDA-MB-231 cells displayed a dose response to paclitaxel in 2D and 3D. IC50 was 1.897µM for 2D monolayer culture and 7.318 µM for 3D culture. ** P<0.05, data mean ± standard deviation, n = 3.

We further characterized the chemotherapy drug sensitivity in Col-Tgel 3D model. Taxol is a cytotoxic chemotherapeutic agent with proven clinical value in breast cancer. MDA-MB-231 cells cultured in 3D scaffolds demonstrated greater resistance than cells in 2D monolayer culture as shown by live/dead cell test (Fig. 6A) with IC_50_ = 1.897 µM for 2D and IC50 = 7.318 µM for 3D culture (Fig. 6B). Anti-mitotic drugs such as taxol were less effective in 3D culture at concentrations that were previously shown to cause apoptosis in monolayer culture.

### Col-Tgel as a carrier for xenograft in animal model

**Figure 7:**
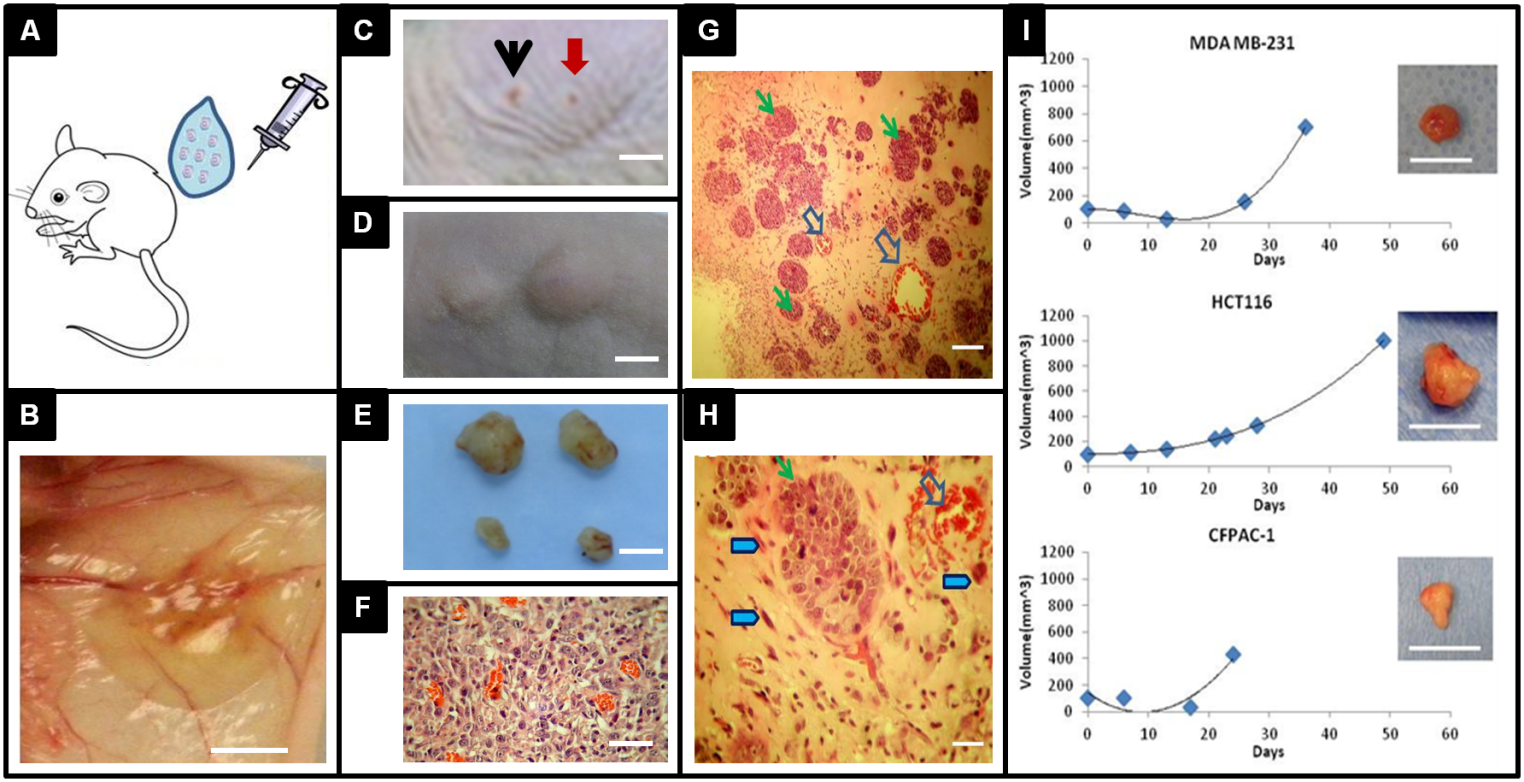
Xenograft tumor induction using Col-Tgel as a carrier. A, Tumor cell and Col-Tgel mixture was injected at the desired site before gel curing. B, Initial tumor formation at the subcutaneous injection site 7-days post injection with gel border clearly defined (scale bar = 2 mm). C–D, Osteosarcoma SaoS-2 cells, 200 µl of Col-Tgel with 1.0×10^6^ cells (dark arrow) or 100 ul of Col-Tgel with 0.5×10^6^ cells (red arrow) were injected subcutaneously in animals (scale bar = 10 mm). Gross tumor formation by observed at day 3 (C) and day 20 (D). E, Retrieved tumors at day 20 exhibited positive correlation between tumor size and initial injection volume (scale bar = 10 mm). F, Histological micrograph of Osteosarcoma tumor with vascular invasion (scale bar = 180 µm). G&H, MDA-MB-231 cells formed clusters (green arrows) and blood vessel infiltration (hollow arrows) inside the Col-Tgel after 14 days of injection (G, scale bar = 550 µm). MDA-MB-231 cells (green arrow), host cells (blue arrows), and new blood vessels (hollow arrow) in the microenvironment created by co-delivery of tumor cells with Col-Tgel after 14 days of injection (H, scale bar = 180 µm). I, Tumor formation curves of different cancer cells using Col-Tgel as carrier (MDA-MD-231, HCT116 and CFPAC-1, scale bar = 10 mm). The plot is a representative value of six tumors.

Xenograft tumors were induced using Col-Tgel as a carrier (Fig. 7A). Gel formed insoluble hydrogel subcutaneously at the injection site with tumor cells embedded. As shown in Fig. 7B, Gel structure was still clearly defined even after 7 days injection and blood vessels invasion was observed. Moreover, the size of tumors could be controlled by the injection volume of gel tumor (same cell density), where the osteosarcoma size by 200 µl of gel tumor was significantly larger than 100 µl after 28 days injection (Fig. 7 C–F). Two injection sides were close but tumor final mass did not fuse or intervene to each other. This observation suggested that the gel were capable to localized cell delivery. Importantly, early stage of tumor development and progression could be studies in the injected semi-enclosed gel environment. Histomicrograph of 7 day post-injection of breast cancer cell MDA-MB-231 clearly showed that tumor cells assembled into clusters ranging from 100 to 500 µm and disperse in the gel unevenly, where the center of gel had less clusters with smaller size while the peripheral region had larger and denser tumor cluster formation, similar to *in vitro* observations (Fig. 7 G–H). Interestingly, blood vessels clearly infiltrated into the gel at day 7 in response to tumor cells, providing nutrients together with host cells such as immune cells, inflammatory cells and mesenchymal stem cells to the tumor environment (Fig. 7 H, hollow arrow and blue arrow). Tumor development induced by Col-Tgel as a carrier was also tested for other tumor cells including breast cancer cell MDA-MB-231, colon cancer cell HCT116, and pancreatic cancer cell CFPAC-1. Representative tumor formation curves were shown in (Fig. 7I). Taken together, this gel carrier bioengineered tumor model may provide a tool box to study the orchestrated tumor formation and progression of events including types of cell participation, spatiotemporal signals molecules exchange between cells, and extracellular matrix protein deposition and degradation.

## Discussion

Col-Tgel is a tailorable collagen-based remodelable hydrogel system able to induce spheroid formation without the use of time and labor intensive protocols such as the hanging drop and linker-engineered method, sophisticated equipment like rotating wall vessel bioreactors, or special handling temperature to prevent self-assembly [35].

*In vitro* 3D culture systems to induce spheroid tumor formation using synthetic, natural or hybrid materials have been extensively attempted [11], [12], [36]. Biocompatible materials such as agarose, methylcellulose, PMMA, PEG are structurally suitable to provide support for tumor spheroid formation, however, they lack cell adhesion and enzyme cleavage sites correlating *in vivo* tissue [5], [37], [38]. Previous studies reported that MDA-MB-231 cells failed to form spheroids and lacked E-cadherin expression when grown on semi-solid methylcellulose [7], [39]–[41], or in round bottom culture well coated with a poly-HEMA [42].These findings suggest that extracellular matrix, such as collagen fragments in Col-Tgel, are important for tumor spheroid formation. Invascu's work [43] demonstrated that type I collagen, but not fibronectin or type IV collagen, not only enhances, but also participates in MDA-MB-231 spheroid formation. Therefore, a biologically functional 3D scaffold is very crucial to simulate the tumor microenvironment. 3D scaffolds fabricated from ECM substrates such as Matrigel, type I collagen, laminin, and fibronectin are cell attachable and remodelable and are ideal materials to construct a tumor tissue scaffold. However, when used in their native purified form, they are unable to provide the wide spectrum of rigidity necessary to mimic normal and pathological conditions [44]. By using collagen peptides with an enzymatic crosslinking technique, Col-Tgel overcomes these limitations and appears suitable for *in vitro* and *in vivo* tumor engineering. Importantly, Col-Tgel offers handling flexibility through controlling the 3D construct size, shape, concentration, and crosslinking rate to achieve structural heterogeneity resemble *in vivo* tumor environment on many aspects, including nutrition diffusion and pH gradients, hypoxia environment, and mechanical restriction. By modulating these parameters according to the tumor progression state, it is possible to bioengineer 3D tumor *in vitro* to closely resemble cancer cells growing in the *in vivo* environment.

Due to the transparent property of the Col-Tgel, we established imaging based assays to monitor cluster formation, cell morphology, delivery of chemotherapies, and cell migration and invasion across 3D constructs in real-time using optical or fluorescence microscopy. Our results suggested that spheroid tumors in the Col-Tgel closely resemble the avascular tumor nodular appearance, contained a necrotic core and proliferative rim in the outer layer, and mimicked the highly preoperative tumor cells located near nutrient rich capillaries *in vivo* [7]. Overall, Col-Tgel 3D architecture presents physiologically relevant characteristics of tumor cells and features simple and easy operation protocols to examine the multiple aspects of cancers.

Col-Tgel is able to easily manipulate and implement a wide range of stiffness by altering the gel concentration and crosslinking units [45]–[49]. Recent studies indicates the powerful influence of biophysical properties, such as rigidity, porosity, density and geometry on cell fates [50]. Thus, physical stiffness of tumor environment may be simulated in an *in vitro* 3D setting to study cell proliferation, differentiation, apoptosis, senescence, and invasion behaviors by tailoring gel formulation [51]–[53]. To more accurately reproduce the tissue specific microenvironment, the matrix composition may be altered by adding different types of extracellular matrices into the Col-Tgel platform, such as various types of collagen (I-IV), adhesion molecules such as laminin, vetronectin, and fibronectin, and proteoglycans and glycoproteins [54]–[56]. For example, pancreatic tumor surrounds by dense fibrillar collagen while brain tumors generates a more amorphous matrix such as hyaluronic acid [57]–[62].

The tumor stroma microenvironment comprises fibroblasts, adipocytes, inflammatory cells such as lymphocytes and macrophages and lymphatic and blood capillaries including pericytes and endothelial cells [63]. Therefore, cancer progression and metastasis depends on the crosstalk within the microenvironments [64]–[67]. However, tumor cells interactions with the extracellular matrix, other cell types, or the immune system is scant or completely absent in 2D monolayer culture. The 3D Col-Tgel system provides a platform for spatial organization of tissues and cell-cell interactions. In our study, we tested co-culture of tumor cells with bone marrow mesenchymal stem cells in a 3D gel. Regarding cell morphologies, the H&E staining demonstrated that both cell types preserve their phenotypic traits. Cancer cells maintained their epithelial morphology and formed spheroids, whereas MSC showed their typical spindle-shaped morphology. We also observed that the two types of cells formed chimera spheroids when labeled with different fluorescence probes, the exact cause and effect of such interactions still needs to be elucidated. Thus, it is possible to recreate some of the *in vivo* tumor niches under highly controlled and reproducible fashions to study tumor cell morphology, phenotype, metabolism and invasion *in vitro* by using co-cultures.

Host cells infiltration into the 3D gel system was observed when delivering cancer cell for xenograft tumor induction. Col-Tgel forms a semi-enclosed system to prevent cancer cell diffusion, in the meanwhile, the cured gel acts as extracellular matrix to support surrounding cells infiltration and migration as they responding to the tumor cell signals. As a result, multicellular tumoroids are formed in situ followed by ECM remodeling at the injection site. We observed that angiogenesis occurred within 7 days, tumor nodular formation within 14 days and mature tumor development in 21–28 days. This new bioengineering tumor will provide us an opportunity to study early stage host cell response by characterizing spatial temporal events of host cell populations and signals exchange, to gain insight of cell-cell communication and their contribution to tumor progression.

In conclusion, we developed a new bioengineering tumor model by using 3D ECM derived peptides in multiple well plates. It is easy to reproducible and cost-effective. The 3D model recapitulates key features of tumors *in vivo* including cell-cell interaction, cell-matrix interactions, and multicellualr architecture. This technology allows creating uniform and highly-reproducible bioengineering tumor for high-content screening of anticancer drugs.

## References

[1] Langley RR, Fidler IJ (2011) The seed and soil hypothesis revisited – The role of tumor – stroma interactions in metastasis to different organs. International Journal of Cancer 128: 2527–2535. 21365651 10.1002/ijc.26031 PMC3075088

[2] Yamada KM, Cukierman E (2007) Modeling tissue morphogenesis and cancer in 3D. Cell 130: 601–610. 17719539 10.1016/j.cell.2007.08.006

[3] Stroock AD, Fischbach C (2010) Microfluidic culture models of tumor angiogenesis. Tissue Engineering Part A 16: 2143–2146. 20214470 10.1089/ten.tea.2009.0689 PMC2947929

[4] Baker BM, Chen CS (2012) Deconstructing the third dimension–how 3D culture microenvironments alter cellular cues. Journal of Cell Science 125: 3015–3024. 22797912 10.1242/jcs.079509 PMC3434846

[5] Fischbach C, Chen R, Matsumoto T, Schmelzle T, Brugge JS, et al (2007) Engineering tumors with 3D scaffolds. Nature Methods 4: 855–860. 17767164 10.1038/nmeth1085

[6] Kim JB (2005) Three-dimensional tissue culture models in cancer biology. Seminar in Cancer Biology 15: 365–377. 10.1016/j.semcancer.2005.05.002 15975824

[7] Nyga A, Cheema U, Loizidou M (2011) 3D tumour models: novel in vitro approaches to cancer studies. Journal of cell communication and signaling 5: 239–248. 21499821 10.1007/s12079-011-0132-4 PMC3145874

[8] Fischbach C, Kong HJ, Hsiong SX, Evangelista MB, Yuen W, et al (2009) Cancer cell angiogenic capability is regulated by 3D culture and integrin engagement. Proceedings of the National Academy of Sciences 106: 399–404. 10.1073/pnas.0808932106 PMC2626714 19126683

[9] Ghajar CM, Bissell MJ (2010) Tumor engineering: the other face of tissue engineering. Tissue Engineering Part A 16: 2153–2156. 20214448 10.1089/ten.tea.2010.0135 PMC2947934

[10] Constantinou AI, Krygier AE, Mehta RR (1998) Genistein induces maturation of cultured human breast cancer cells and prevents tumor growth in nude mice. The American journal of clinical nutrition 68: 1426S–1430S. 9848511 10.1093/ajcn/68.6.1426S

[11] Ong S-M, Zhao Z, Arooz T, Zhao D, Zhang S, et al (2010) Engineering a scaffold-free 3D tumor model for in vitro drug penetration studies. Biomaterials 31: 1180–1190. 19889455 10.1016/j.biomaterials.2009.10.049

[12] Timmins NE, Nielsen LK (2007) Generation of multicellular tumor spheroids by the hanging-drop method. Tissue Engineering: Springer. 141–151. 10.1007/978-1-59745-443-8_8 18085207

[13] Albini A, Iwamoto Y, Kleinman H, Martin G, Aaronson S, et al (1987) A rapid in vitro assay for quantitating the invasive potential of tumor cells. Cancer Research 47: 3239–3245. 2438036

[14] Passaniti A, Isaacs JT, Haney JA, Adler SW, Cujdik TJ, et al (1992) Stimulation of human prostatic carcinoma tumor growth in athymic mice and control of migration in culture by extracellular matrix. International Journal of Cancer 51: 318–324. 1568798 10.1002/ijc.2910510224

[15] Miller BE, Miller FR, Heppner GH (1985) Factors affecting growth and drug sensitivity of mouse mammary tumor lines in collagen gel cultures. Cancer Research 45: 4200–4205. 4028010

[16] Doillon CJ, Gagnon E, Paradis R, Koutsilieris M (2004) Three-dimensional culture system as a model for studying cancer cell invasion capacity and anticancer drug sensitivity. Anticancer Research 24: 2169–2178. 15330157

[17] Gurski LA, Jha AK, Zhang C, Jia X, Farach-Carson MC (2009) Hyaluronic acid-based hydrogels as 3D matrices for *in vitro* evaluation of chemotherapeutic drugs using poorly adherent prostate cancer cells. Biomaterials 30: 6076–6085. 19695694 10.1016/j.biomaterials.2009.07.054 PMC2782556

[18] Kibbey MC, Grant DS, Kleinman HK (1992) Role of the SIKVAV site of laminin in promotion of angiogenesis and tumor growth: an in vivo Matrigel model. Journal of the National Cancer Institute 84: 1633–1638. 1279183 10.1093/jnci/84.21.1633

[19] Kessenbrock K, Plaks V, Werb Z (2010) Matrix metalloproteinases: regulators of the tumor microenvironment. Cell 141: 52–67. 20371345 10.1016/j.cell.2010.03.015 PMC2862057

[20] Sung S-Y, Hsieh C-L, Wu D, Chung LW, Johnstone PA (2007) Tumor microenvironment promotes cancer progression, metastasis, and therapeutic resistance. Current problems in cancer 31: 36–100. 17362788 10.1016/j.currproblcancer.2006.12.002

[21] Szot CS, Buchanan CF, Freeman JW, Rylander MN (2011) 3D in vitro bioengineered tumors based on collagen I hydrogels. Biomaterials 32: 7905–7912. 21782234 10.1016/j.biomaterials.2011.07.001 PMC3229258

[22] Kuwahara K, Yang Z, Slack GC, Nimni ME, Han B (2009) Cell delivery using an injectable and adhesive transglutaminase–gelatin gel. Tissue Engineering Part C: Methods 16: 609–618. 10.1089/ten.TEC.2009.0406 19757996

[23] Kuwahara K, Fang JY, Yang Z, Han B (2011) Enzymatic crosslinking and degradation of gelatin as a switch for bone morphogenetic protein-2 activity. Tissue Engineering Part A 17: 2955–2964. 21882896 10.1089/ten.tea.2011.0290

[24] Fang J, Yang Z, Tan S, Tayag C, Nimni ME, et al (2014) Injectable gel graft for bone defect repair. Regenerative medicine 9: 41–51. 24351005 10.2217/rme.13.76 PMC9302184

[25] Trédan O, Galmarini CM, Patel K, Tannock IF (2007) Drug resistance and the solid tumor microenvironment. Journal of the National Cancer Institute 99: 1441–1454. 17895480 10.1093/jnci/djm135

[26] Ungefroren H, Sebens S, Groth S, Gieseler F, Fandrich F (2011) The Src family kinase inhibitors PP2 and PP1 block TGF-beta1-mediated cellular responses by direct and differential inhibition of type I and type II TGF-beta receptors. Current Cancer Drug Targets 11: 524–535. 21395548 10.2174/156800911795538075

[27] Su S, Chang Y, Andreu-Vieyra C, Fang J, Yang Z, et al (2012) miR-30d, miR-181a and miR-199a-5p cooperatively suppress the endoplasmic reticulum chaperone and signaling regulator GRP78 in cancer. Oncogene. 10.1038/onc.2012.483 PMC3787795 23085757

[28] Masood R, Kumar SR, Sinha UK, Crowe DL, Krasnoperov V, et al (2006) EphB4 provides survival advantage to squamous cell carcinoma of the head and neck. International Journal of Cancer 119: 1236–1248. 16615113 10.1002/ijc.21926

[29] Gordon EM, Michael S, Kundu RK, Han B, Andrades J, et al (1997) Capture and Expansion of Bone Marrow-Derived Mesenchymal Progenitor Cells with a Transforming Growth Factor-β1–von Willebrand's Factor Fusion Protein for Retrovirus-Mediated Delivery of Coagulation Factor IX. Human Gene Therapy 8: 1385–1394. 9295133 10.1089/hum.1997.8.11-1385

[30] Ahearne M, Yang Y, Liu K (2008) Mechanical characterisation of hydrogels for tissue engineering applications. Topics in tissue Engineering 4: 1–16.

[31] Yang Y, Bagnaninchi PO, Ahearne M, Wang RK, Liu K-K (2007) A novel optical coherence tomography-based micro-indentation technique for mechanical characterization of hydrogels. Journal of the Royal Society Interface 4: 1169–1173. 10.1098/rsif.2007.1044 PMC2396212 17472904

[32] Chau D, Collighan RJ, Verderio EA, Addy VL, Griffin M (2005) The cellular response to transglutaminase-cross-linked collagen. Biomaterials 26: 6518–6529. 15927250 10.1016/j.biomaterials.2005.04.017

[33] Davis GE, Bayless KJ, Davis MJ, Meininger GA (2000) Regulation of tissue injury responses by the exposure of matricryptic sites within extracellular matrix molecules. The American Journal of Pathology 156: 1489–1498. 10793060 10.1016/S0002-9440(10)65020-1 PMC1876929

[34] Kilian KA, Bugarija B, Lahn BT, Mrksich M (2010) Geometric cues for directing the differentiation of mesenchymal stem cells. Proceedings of the National Academy of Sciences 107: 4872–4877. 10.1073/pnas.0903269107 PMC2841932 20194780

[35] Wulf E, Deboben A, Bautz F, Faulstich H, Wieland T (1979) Fluorescent phallotoxin, a tool for the visualization of cellular actin. Proceedings of the National Academy of Sciences 76: 4498–4502. 10.1073/pnas.76.9.4498 PMC411604 291981

[36] Tilghman RW, Blais EM, Cowan CR, Sherman NE, Grigera PR, et al (2012) Matrix rigidity regulates cancer cell growth by modulating cellular metabolism and protein synthesis. PLoS One 7: e37231. 22623999 10.1371/journal.pone.0037231 PMC3356407

[37] Agastin S, Giang U-BT, Geng Y, DeLouise LA, King MR (2011) Continuously perfused microbubble array for 3D tumor spheroid model. Biomicrofluidics 5: 024110. 10.1063/1.3596530 PMC3124519 21716809

[38] Talukdar S, Mandal M, Hutmacher DW, Russell PJ, Soekmadji C, et al (2011) Engineered silk fibroin protein 3D matrices for *in vitro* tumor model. Biomaterials 32: 2149–2159. 21167597 10.1016/j.biomaterials.2010.11.052

[39] Loessner D, Stok KS, Lutolf MP, Hutmacher DW, Clements JA, et al (2010) Bioengineered 3D platform to explore cell–ECM interactions and drug resistance of epithelial ovarian cancer cells. Biomaterials 31: 8494–8506. 20709389 10.1016/j.biomaterials.2010.07.064

[40] Bradbury P, Fabry B, O'Neill GM (2012) Occupy tissue: The movement in cancer metastasis. Cell adhesion & migration 6: 424–520. 23076050 10.4161/cam.21559 PMC3496680

[41] Fraley SI, Feng Y, Krishnamurthy R, Kim D-H, Celedon A, et al (2010) A distinctive role for focal adhesion proteins in three-dimensional cell motility. Nature cell biology 12: 598–604. 20473295 10.1038/ncb2062 PMC3116660

[42] Iglesias JM, Beloqui I, Garcia-Garcia F, Leis O, Vazquez-Martin A, et al (2013) Mammosphere Formation in Breast Carcinoma Cell Lines Depends upon Expression of E-cadherin. PloS one 8: e77281. 24124614 10.1371/journal.pone.0077281 PMC3790762

[43] Ivascu A, Kubbies M (2006) Rapid generation of single-tumor spheroids for high-throughput cell function and toxicity analysis. Journal of biomolecular screening 11: 922–932. 16973921 10.1177/1087057106292763

[44] Ivascu A, Kubbies M (2007) Diversity of cell-mediated adhesions in breast cancer spheroids. International journal of oncology 31: 1403–1414. 17982667

[45] Evans CL, Abu-Yousif AO, Park YJ, Klein OJ, Celli JP, et al (2011) Killing hypoxic cell populations in a 3D tumor model with EtNBS-PDT. PLoS ONE 6: e23434. 21876751 10.1371/journal.pone.0023434 PMC3158086

[46] Büchler P, Reber HA, Lavey RS, Tomlinson J, Büchler MW, et al (2004) Tumor hypoxia correlates with metastatic tumor growth of pancreatic cancer in an orthotopic murine model 1. Journal of Surgical Research 120: 295–303. 15234226 10.1016/j.jss.2004.02.014

[47] Vaupel P, Mayer A (2007) Hypoxia in cancer: significance and impact on clinical outcome. Cancer and Metastasis Reviews 26: 225–239. 17440684 10.1007/s10555-007-9055-1

[48] Brammer I, Zywietz F, Jung H (1979) Changes of histological and proliferative indices in the Walker carcinoma with tumour size and distance from blood vessel. European Journal of Cancer (1965) 15: 1329–1336. 10.1016/0014-2964(79)90109-9 527625

[49] Rajendran JG, Krohn KA (2005) Imaging hypoxia and angiogenesis in tumors. Radiologic Clinics of North America 43: 169–187. 15693655 10.1016/j.rcl.2004.08.004

[50] Crescenzi V, Francescangeli A, Taglienti A (2002) New gelatin-based hydrogels via enzymatic networking. Biomacromolecules 3: 1384–1391. 12425680 10.1021/bm025657m

[51] Barcus CE, Keely PJ, Eliceiri KW, Schuler LA (2013) Stiff collagen matrices increase tumorigenic prolactin signaling in breast cancer cells. Journal of Biological Chemistry 288: 12722–12732. 23530035 10.1074/jbc.M112.447631 PMC3642318

[52] Baker EL, Lu J, Yu D, Bonnecaze RT, Zaman MH (2010) Cancer cell stiffness: integrated roles of three-dimensional matrix stiffness and transforming potential. Biophysical Journal 99: 2048–2057. 20923638 10.1016/j.bpj.2010.07.051 PMC3042573

[53] Hakkinen KM, Harunaga JS, Doyle AD, Yamada KM (2010) Direct comparisons of the morphology, migration, cell adhesions, and actin cytoskeleton of fibroblasts in four different three-dimensional extracellular matrices. Tissue Engineering Part A 17: 713–724. 20929283 10.1089/ten.tea.2010.0273 PMC3043991

[54] Ma L, Zhou C, Lin B, Li W (2010) A porous 3D cell culture micro device for cell migration study. Biomedical microdevices 12: 753–760. 20455081 10.1007/s10544-010-9429-y PMC2913465

[55] Zaman MH, Trapani LM, Sieminski AL, MacKellar D, Gong H, et al (2006) Migration of tumor cells in 3D matrices is governed by matrix stiffness along with cell-matrix adhesion and proteolysis. Proceedings of the National Academy of Sciences 103: 10889–10894. 10.1073/pnas.0604460103 PMC1544144 16832052

[56] PaszeK MJ, Zahir N, Johnson KR, Lakins JN, Rozenberg GI, et al (2005) Tensional homeostasis and the malignant phenotype. Cancer cell 8: 241–254. 16169468 10.1016/j.ccr.2005.08.010

[57] Zahir N, Lakins JN, Russell A, Ming W, Chatterjee C, et al (2003) Autocrine laminin-5 ligates α6β4 integrin and activates RAC and NFκB to mediate anchorage-independent survival of mammary tumors. The Journal of cell biology 163: 1397–1407. 14691145 10.1083/jcb.200302023 PMC2173718

[58] Pirazzoli V, Ferraris GMS, Sidenius N (2013) Direct evidence of the importance of vitronectin and its interaction with the urokinase receptor in tumor growth. Blood 121: 2316–2323. 23327926 10.1182/blood-2012-08-451187

[59] Mao Y, Schwarzbauer JE (2005) Stimulatory effects of a three-dimensional microenvironment on cell-mediated fibronectin fibrillogenesis. Journal of cell science 118: 4427–4436. 16159961 10.1242/jcs.02566

[60] Chekenya M, Hjelstuen M, Enger PØ, Thorsen F, Jacob AL, et al (2002) NG2 proteoglycan promotes angiogenesis-dependent tumor growth in CNS by sequestering angiostatin. The FASEB Journal 16: 586–588. 11919162 10.1096/fj.01-0632fje

[61] Hendriks J, Planelles L, de Jong-Odding J, Hardenberg G, Pals S, et al (2005) Heparan sulfate proteoglycan binding promotes APRIL-induced tumor cell proliferation. Cell Death & Differentiation 12: 637–648. 15846369 10.1038/sj.cdd.4401647

[62] Saverio B, Pierpaola D, Serenella A, Cesare C, Bruno M, et al (2000) Tumor progression is accompanied by significant changes in the levels of expression of polyamine metabolism regulatory genes and clusterin (sulfated glycoprotein 2) in human prostate cancer specimens. Cancer Research 60: 28–34. 10646846

[63] Ananthanarayanan B, Kim Y, Kumar S (2011) Elucidating the mechanobiology of malignant brain tumors using a brain matrix-mimetic hyaluronic acid hydrogel platform. Biomaterials 32: 7913–7923. 21820737 10.1016/j.biomaterials.2011.07.005 PMC3159794

[64] Bissell MJ, LaBarge MA (2005) Context, tissue plasticity, and cancer: are tumor stem cells also regulated by the microenvironment? Cancer cell 7: 17. 15652746 10.1016/j.ccr.2004.12.013 PMC2933216

[65] Yu H, Kortylewski M, Pardoll D (2007) Crosstalk between cancer and immune cells: role of STAT3 in the tumour microenvironment. Nature Reviews Immunology 7: 41–51. 10.1038/nri1995 17186030

[66] Allavena P, Sica A, Solinas G, Porta C, Mantovani A (2008) The inflammatory micro-environment in tumor progression: the role of tumor-associated macrophages. Critical reviews in oncology/hematology 66: 1–9. 17913510 10.1016/j.critrevonc.2007.07.004

[67] Whiteside T (2008) The tumor microenvironment and its role in promoting tumor growth. Oncogene 27: 5904–5912. 18836471 10.1038/onc.2008.271 PMC3689267

